# “Silent Suffering”: Affirming Iranian Women's Right to Sexual Health Through a Whatsapp‐Based “Good Enough Sex” Counseling Program: A Parallel‐Group Randomized Controlled Trial

**DOI:** 10.1002/hsr2.73340

**Published:** 2026-10-07

**Authors:** Emdadi‐Babanazr Shahla, Moghaddam‐Tabrizi Fatemeh, Haghighi Mahmonir, Gharaaghaji Asl Rasool

**Affiliations:** ^1^ School of Nursing and Midwifery Urmia University of Medical Sciences Urmia Iran; ^2^ Department of Psychiatry, School of Medicine Razi Hospital Urmia University of Medical Sciences Urmia Iran; ^3^ Department of Community Medicine, School of Medicine Urmia University of Medical Sciences Urmia Iran

**Keywords:** counseling, good enough sex, infertility, infertility stress, sexual satisfaction

## Abstract

**Introduction:**

Infertility profoundly affects women's sexual health and psychological well‐being, particularly in cultures where childbearing defines female identity and sexuality remains a taboo subject. In Iran, no randomized controlled trial has evaluated a culturally adapted digital psychosexual intervention for infertile women.

**Methods:**

This parallel‐group, single‐blind randomized controlled trial was conducted from September 2021 to April 2022 at two major infertility centers in Urmia, Iran. A total of 100 infertile women (aged 25–35 years) were recruited via convenience sampling and then randomly allocated to either a 6‐week WhatsApp‐based Good Enough Sex (GES) counseling program (*n* = 50) or a routine care control group (*n* = 50). Outcomes included infertility‐related stress (Newton Infertility Stress Questionnaire) and sexual satisfaction (Larson's Sexual Satisfaction Questionnaire), assessed at baseline, post‐intervention, and 1‐month follow‐up. Data were analyzed using repeated‐measures ANOVA.

**Results:**

The intervention group showed significant improvements in sexual satisfaction (mean increase: 41.92 points) and significant reductions in infertility‐related stress (mean reduction: 108.90 points) immediately post‐intervention, with effects largely sustained at 1‐month follow‐up (*p* < 0.001 for all). Effect sizes were large (partial η^2^ = 0.52 for sexual satisfaction; partial η^2^ = 0.82 for infertility stress). The control group showed no significant changes.

**Conclusions:**

Among this sample of infertile Iranian women, WhatsApp‐based GES counseling was associated with reduced infertility‐related stress and improved sexual satisfaction. Because the sample was recruited by convenience sampling from two referral centers, was relatively small, used restrictive eligibility criteria, and had only 1‐month follow‐up, these findings should be considered promising preliminary evidence. Larger multicenter randomized trials with more diverse populations and longer follow‐up are needed before conclusions about generalizability can be drawn. If confirmed, this approach may offer a useful adjunct to infertility care in culturally conservative settings.

**Policy Implications:**

In conservative societies where sexual health discussions are stigmatized, infertility clinics may consider integrating WhatsApp‐based GES counseling into routine care. Policymakers could support training providers in this model and digital ethics, and health authorities can leverage this approach to replace online misinformation with evidence‐based education.

**Trial Registration:**

IRCT20210926052593N1, The date of registration: 13‐10‐2021.

**Abbreviation**
GES model: GES Good Enough Sex Model

## 1 Introduction

Infertility is defined by the World Health Organization as the inability to conceive after 12 months of regular, unprotected intercourse [1]. Globally, infertility affects approximately 48 million couples, with prevalence rates varying widely across sociocultural contexts [2]. In Iran, a recent systematic review and meta‐analysis reported a pooled infertility prevalence of 10.5% (95% CI: 8.9%–12.3%), with primary infertility accounting for 10.6% and secondary infertility for 2.6% [3]. However, these epidemiological estimates only partially capture the burden of infertility, because in societies where childbearing is central to female identity and marital stability, the psychosocial impact of infertility far exceeds its biomedical dimensions [4].

Infertility is not merely a medical condition; it is a multidimensional stressor that profoundly affects emotional well‐being, marital relationships, and quality of life [5]. Among the various domains of infertility‐related distress, sexual concerns are consistently reported as one of the most distressing [6]. Women and men with infertility often experience heightened sexual anxiety, reduced sexual desire, and diminished sexual satisfaction [7]. The medically timed intercourse required by many fertility treatments further exacerbates performance pressure and erodes spontaneous sexual intimacy [8]. Consequently, infertile women report significantly lower sexual satisfaction than fertile counterparts [8].

From a biopsychosocial perspective, infertility‐related sexual dysfunction can be understood through the lens of the dual control model [9] and the interpersonal exchange model of sexual satisfaction [10]. The dual control model proposes that individual differences in sexual excitation and inhibition significantly affect sexual functioning; importantly, individuals from cultures where sexual topics are highly taboo may have heightened sexual inhibition proneness [9]. The Interpersonal Exchange Model of Sexual Satisfaction conceptualizes sexual satisfaction as determined by the balance between sexual rewards and costs, relationship satisfaction, and partner dynamics [10]. In traditional societies, where discussing sexual concerns is taboo, these cognitive and interpersonal pathways to sexual satisfaction are particularly compromised because couples lack the language and social permission to address sexual issues openly [11, 12]. The Good Enough Sex (GES) model, introduced by Metz and McCarthy [13], offers a framework that directly addresses these cognitive and interpersonal pathways.

Iranian society exemplifies such a context. In Iran, sexuality is a highly sensitive and stigmatized subject rarely discussed in public, within families, or even between spouses [14]. Healthcare providers themselves avoid initiating sexual health conversations, fearing moral judgment and professional repercussions [14, 15]. This institutionalized silence creates a paradoxical situation: although infertile women experience significant sexual distress, they are often reluctant to seek face‐to‐face counseling due to shame, modesty, and fear of being labeled as morally deviant [16]. Qualitative research from Iran has shown that women frequently internalize infertility as a personal failure, and they resort to secrecy and social withdrawal rather than disclosing sexual problems [17]. In fact, a recent phenomenological study reported that many infertile Iranian women described their sexual lives as “silent suffering” that they had never verbalized to anyone [18].

A promising psychosexual intervention that directly addresses these challenges is the Good Enough Sex (GES) model developed by Metz and McCarthy [19]. The GES model shifts the focus from performance‐based, intercourse‐centric expectations toward pleasure‐oriented, flexible, and intimate sexual activity. Its core principles include [1]: replacing unrealistic sexual perfectionism with “good enough” experiences [2]; integrating sexuality across the life span, acknowledging fluctuations due to stress or illness [3]; emphasizing mutual pleasure rather than orgasm as the sole goal; and [4] fostering non‐coercive, affectionate touch. By destigmatizing normal sexual variation and promoting non‐coercive, affectionate communication, the GES framework directly targets the heightened sexual inhibition and reduced sexual rewards common in infertile couples. The model has been successfully applied to couples coping with chronic illness, cancer, and sexual dysfunction, but its application to infertility especially in non‐Western cultures remains underexplored [13, 20].

Crucially, the GES model's emphasis on verbal communication and gradual exposure aligns well with digital delivery. WhatsApp was selected as the delivery platform for three interrelated reasons. First, it is the most widely used messaging application among Iranian women across all socioeconomic strata, ensuring near‐universal familiarity and eliminating technical barriers to participation. Second, WhatsApp allows anonymous participation (via pseudonyms and dedicated SIM cards), which is particularly vital in a culture where face‐to‐face discussion of sex is taboo; it provides a safe, asynchronous, and private space for psychosexual education without the shame associated with clinical waiting rooms. Third, from a practical healthcare perspective, WhatsApp requires no additional infrastructure, training, or cost, making it a highly scalable and sustainable option within routine infertility care settings [21]. Women who would never speak about sexual issues in a clinic waiting room may feel comfortable typing questions anonymously in a WhatsApp group [21]. A growing body of evidence supports the feasibility of WhatsApp‐based interventions for sensitive health topics, including mental health, diabetes self‐management, and even sexual health education among adolescents [22, 23].

However, no previous study has applied a WhatsApp‐delivered GES intervention to infertile women in a Middle Eastern setting.

Thus, a clear gap exists: despite the high prevalence of infertility‐related sexual distress in Iran, and despite the theoretical suitability of the GES model and digital delivery, no randomized controlled trial has tested a WhatsApp‐based GES counseling program for this population. This study was designed to fill that gap. Importantly, we conceptualize the intervention not only as a therapeutic tool but also as a culturally sensitive opportunity to break the silence around sexuality to legitimize sexual concerns as a normal part of infertility care, and to provide women with a discreet channel to reclaim their sexual well‐being without violating cultural norms of modesty.

Therefore, the present study aimed to evaluate the effect of a WhatsApp‐based sexual counseling program using the Good Enough Sex model on infertility‐related stress and sexual satisfaction among infertile women attending infertility centers in Urmia, Iran, using a parallel‐group randomized controlled trial design. We hypothesized that, compared to the routine care control group, the GES‐based intervention would significantly reduce infertility‐related stress (across all its subscales: social concerns, sexual concerns, communication concerns, need for parenthood, and rejection of life without children) and significantly improve sexual satisfaction (including desire, attitude, quality of life, and adjustment), with these effects being sustained at the 1‐month follow‐up.

## 2 Methods

### 2.1 Study Design

This was a parallel‐group, single‐blind, randomized controlled trial. Eligible infertile women (recruited through convenience sampling) were randomly assigned to either the intervention group (WhatsApp‐based GES counseling plus routine care) or the control group (routine care only). Outcome assessments were conducted at three time points: baseline (T0), immediately post‐intervention (T1, 6 weeks), and 1 month post‐intervention (T2, 10 weeks after baseline). All questionnaires were completed in person at the infertility clinics.

### 2.2 Participants

Inclusion criteria were: (a) primary infertility; (b) married and living with spouse; (c) age 25–35 years; (d) no intention to use donor eggs, embryos, surrogacy, or adoption; (e) no major psychiatric disorder at the time of the study (initial diagnosis by a psychiatrist) and no use of psychiatric medications; (f) no smoking (cigarette or hookah), no alcohol consumption, and no systemic diseases (e.g., diabetes) based on medical history; (g) total score < 23 on the General Health Questionnaire‐28 (GHQ‐28); (h) not receiving any other sexual education or counseling; (i) self‐reported absence of sexual dysfunction in the spouse; (j) ability to be contacted for at least 4 months; and (k) having a smartphone with internet access. Exclusion criteria were: (a) any physical or mental illness, accident, or psychological trauma affecting sexual performance; (b) pregnancy during the study; (c) withdrawal from the WhatsApp group; or (d) participating in other counseling sessions outside the study program.

## 3 Assessments

**Demographic Questionnaire** collected: age of patient and spouse, education level (patient and spouse), occupation (patient and spouse), adequacy of family income, duration of infertility diagnosis, treatment length, and cause of infertility.

**General Health Questionnaire (GHQ‐28)** was used to screen mental health. It consists of four subscales: somatic symptoms (items 1–7), anxiety/insomnia (items 8–14), social dysfunction (items 15–21), and severe depression (items 22–28). Items are rated on a 4‐point Likert scale (0–3). A score ≥ 23 indicates probable mental health disorder [24]; women scoring > 23 were excluded. The validity and reliability of the GHQ‐28 have been confirmed in Iranian populations. The Persian version has demonstrated good internal consistency (Cronbach's α = 0.78–0.94), split‐half reliability (0.89–0.90), and test‐retest reliability (0.58) [25, 26, 27]. Criterion validity with psychiatric diagnosis was high (sensitivity 0.80–0.83, specificity 0.76–0.99) [26, 27].

**Larson's Sexual Satisfaction Questionnaire** (LSSQ) contains 25 items rated on a 5‐point Likert scale (never to always), total score 25–125. Higher scores indicate higher sexual satisfaction: < 50 = low, 51–100 = moderate, > 100 = high [28]. Its Persian version has validated reliability and validity [29, 30].

**Newton Infertility Stress Questionnaire (NISQ)** is a 46‐item tool with five subscales: social concerns (10 items), sexual concerns (8 items), communication concerns (10 items), rejection of life without children (8 items), and need for parenthood (10 items). Items are rated on a 6‐point Likert scale (total 46–276); higher scores indicate more stress [31]. The Persian version, validated by Alizadeh et al. (2005), yielded Cronbach's alphas of 0.78, 0.77, 0.78, 0.75, and 0.84 for the subscales, and 0.91 for total stress [32].

## 4 Procedures and Randomization

The trial was conducted from September 2021 to April 2022 at two major infertility centers in Urmia, Iran: Kowsar Women's Comprehensive Educational and Medical Center (the sole public referral infertility center in West Azerbaijan Province, covering the entire northwest region of Iran) and Dr. Tizro Infertility Center (the largest private infertility center in the province, which also accepts patients from neighboring provinces). The selection of these two centers ensured a representative sample of infertile women from a wide geographic catchment area, encompassing both public and private healthcare sectors.

First, convenience sampling was used for recruitment to screen 168 infertile women; of these, 100 met the eligibility criteria and provided written informed consent (Figure 1). The enrollment and baseline assessments were conducted by a trained research midwife (SH.E.B). A simple random allocation (1:1) was performed using a free online randomization service (https://www.sealedenvelope.com/simple-randomiser). The allocation sequence was generated by an independent research assistant who had no contact with the participants and was not involved in the enrollment process. After completing baseline assessments, the research midwife assigned each participant the next sequential number in the order of enrollment, and the online system automatically revealed the group allocation based on the pre‐generated sequence. This ensured that the research midwife conducting the enrollment and assessments was blind to the upcoming allocation, thereby maintaining allocation concealment and minimizing selection bias. To provide a complete picture of the blinding status across the trial [1]: participants were not blinded, as the educational nature of the intervention made it impossible to conceal whether they were receiving the GES counseling or routine advice [2]; the counselor delivering the program was also not blinded for the same reason [3]; the outcome assessor, who administered the follow‐up questionnaires in person at the clinics, was kept entirely unaware of group allocation throughout the study; and [4] the statistician who performed the final data analysis received only coded data with no group identifiers and remained blinded to group assignments until all analyses were completed. This approach minimizes detection bias (via blinded outcome assessment) and analysis bias (via blinded data analysis), while acknowledging the inherent impossibility of blinding participants and providers in behavioral interventions.

**Figure 1:**
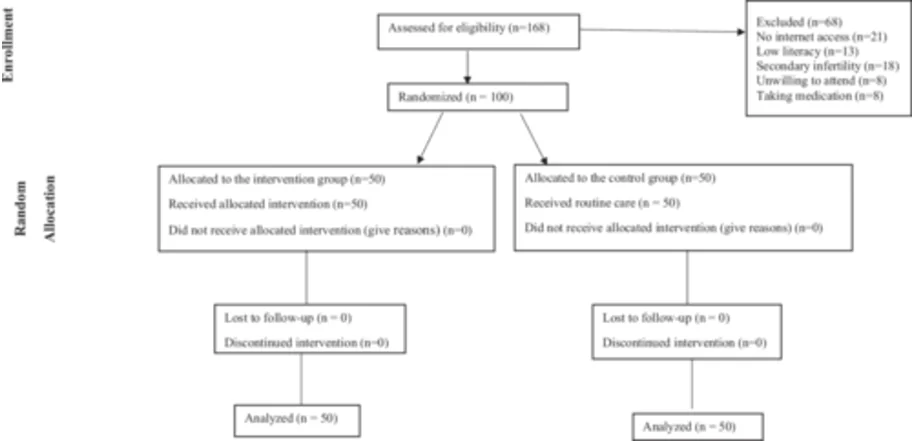
CONSORT flow diagram of participant recruitment and randomization. A total of 168 infertile women were screened for eligibility; 68 were excluded (21 lacked internet access, 13 had low literacy, 18 had secondary infertility, 8 were unwilling to participate, and 8 were taking medication for medical issues). The remaining 100 eligible participants were randomly allocated to the intervention group (WhatsApp‐based GES counseling plus routine care, *n* = 50) or the control group (routine care only, *n* = 50). All 50 participants in each group completed the 6‐week intervention/follow‐up period with no attrition. Data from all 100 participants were included in the final analysis (intention‐to‐treat). GES, Good Enough Sex.

After obtaining written informed consent, all participants completed the baseline (pretest) questionnaires (demographics, NISQ, LSSQ). Then WhatsApp groups were created for the intervention group, which received the 6‐week GES program (Table 1) in addition to routine infertility advice. The control group received only routine infertility counseling via WhatsApp, including topics such as timing of intercourse, personal hygiene, and general health care without any GES‐specific content. After completing the final follow‐up questionnaires, participants in the control group were offered the full GES intervention as an ethical consideration. To respect privacy and anonymity, participants were encouraged to use pseudonyms in the WhatsApp groups, and the research team provided 20 SIM cards to those unwilling to reveal their personal phone numbers. Post‐test questionnaires were completed immediately after the 6‐week intervention and again at 1‐month follow‐up.

**Table 1:** WhatsApp‐based GES Counseling Program (6 Weekly Sessions).

Session	Title	Core GES focus (dimensions & key principles)	WhatsApp activities (text/audio/private chat)	Homework	Practical details: duration & adherence
1	Breaking the Silence: Understanding Sexual Taboos	**Dimensions 1 & 2**: Identifying couples' sexuality; correcting fears, shame, and negative attitudes	Anonymous group message: *“One sexual fear I have never shared…”* + psychoeducational text on normalizing concerns	Write daily: one sexual worry and one positive belief about intimacy (private note)	**Sync session:** 30–45 min voice call; **Daily:** 20–30 min reading/writing; **Adherence:** counselor reviews notes weekly, reminders sent within 48 h if no engagement
2	Realistic Expectations About Sex	**Dimensions 3 & 4**: Realistic expectations versus perfectionism; sexuality as a lifelong process	Group discussion: *“What unrealistic messages about sex have we learned from media?”* + short audio on “good enough sex”	List 5 unrealistic beliefs and replace each with a realistic, self‐compassionate alternative	**Sync session:** 30–45 min voice call; **Daily:** 20–30 min; **Adherence:** homework submissions tracked, personalized reminders for non‐submission
3	Physical Health, Relaxation, and Pleasure	**Dimensions 5 & 6**: Healthy behaviors (sleep, exercise); relaxation and pleasure as equally important	Guided breathing exercise (voice note); sharing a non‐coital pleasurable activity with partner (private chat)	Practice a 10‐min sensual touch exercise without intercourse; report feelings	**Sync session:** 30–45 min; **Daily:** 20–30 min; **Adherence:** daily participation logs reviewed, voice note submissions tracked
4	Managing Performance Pressure and Fear of Failure	**Dimensions 7 & 8**: Overcoming fear of failure; flexible sexual experiences; abandoning perfect performance	Group discussion: *“How does timed intercourse affect my desire?”* + counselor's tips on reducing performance anxiety	Design a “low‐pressure sexual scenario” (no goal of orgasm/pregnancy) and try it once	**Sync session:** 30–45 min; **Daily:** 20–30 min; **Adherence:** attendance at sync sessions recorded, homework reviewed within 48 h
5	Gender Differences and the Five Purposes of Sex	**Dimensions 9 & 10**: Gender differences; integrating five purposes: reproduction, tension reduction, pleasure, self‐esteem, intimacy	Private reflection: *“Which purpose matters most to me right now?”* + optional group sharing	Discuss with partner the five purposes and identify one non‐reproductive purpose to focus on	**Sync session:** 30–45 min; **Daily:** 20–30 min; **Adherence:** reflection notes reviewed, partner discussion verified via private chat
6	Integrating Sex into Real Life – Personalizing Sexuality	**Dimensions 11 & 12**: Sex as playful, spiritual, and integrated into daily life; emotional intimacy as ultimate focus	Designing a personalized sexual scenario (shared privately with counselor) + group sharing of lessons learned	Perform the personalized scenario; complete post‐intervention questionnaires	**Sync session:** 30–45 min; **Daily:** 20–30 min; **Adherence:** final scenario reviewed, post‐test completion tracked

*Note:* The program was adapted from the original Good Enough Sex (GES) model by Metz & McCarthy (2007) and modified for asynchronous WhatsApp delivery in a conservative, high‐stigma cultural context. Each week, participants received a structured combination of psychoeducational texts, group discussion prompts, anonymous sharing opportunities, voice notes, and private counseling messages. Homework tasks were reviewed and reinforced by the counselor via private chat.

## 5 Sample Size

Sample size was calculated using G Power software (version 3.1) based on a previous study that reported sexual satisfaction means and standard deviations in a similar population [33]. The primary outcome used as the basis for the calculation was the total score of the Larson Sexual Satisfaction Questionnaire at post‐intervention (immediately after the 6‐week program). Assuming a two‐sided significance level (α = 0.05), power of 80%, and mean differences between the intervention and control groups (intervention: 93.67 ± 8.07; control: 89 ± 7.89), the required sample size was 42 per group. This calculation was based on a between‐group comparison at post‐intervention. Accounting for 20% attrition, we enrolled 50 participants per group (total *N* = 100).

## 6 Ethics Approval and Trial Registration

The study protocol was approved by the Ethics Committee of the Urmia University of Medical Sciences. All participants provided written informed consent. The trial was registered in the Iranian Registry of Clinical Trials (IRCT). Participants were assured of their right to withdraw at any time without affecting their medical care.

## 7 Intervention

The intervention was a 6‐week structured program based on the 12 dimensions of the Good Enough Sex (GES) model, delivered via WhatsApp (Table 1). The 12 dimensions were distributed across the 6 weekly sessions, with each session covering two core dimensions: Session 1 (Dimensions 1–2), Session 2 (Dimensions 3–4), Session 3 (Dimensions 5–6), Session 4 (Dimensions 7–8), Session 5 (Dimensions 9–10), and Session 6 (Dimensions 11–12).

To enhance replicability, additional practical details are provided here. Although the program was primarily delivered asynchronously via text, voice notes, and video clips, each weekly module included a structured 30‐ to 45‐min synchronous group discussion via WhatsApp voice calls, followed by asynchronous reading and homework activities requiring approximately 20–30 min of daily engagement. All participants received the same core weekly homework assignments (as outlined in Table 1), though personalized adjustments were offered during private chats with the counselor to accommodate individual barriers or sensitivities. Adherence was monitored systematically: the research counselor tracked daily participation logs, reviewed submitted homework notes, and recorded attendance at synchronous group discussions. Participants who did not submit their homework or engage in group discussions within 48 h received a personalized reminder via private message. This monitoring revealed that over 90% of participants completed at least 80% of the weekly assignments, consistent with the 100% retention rate observed in the intervention group.

The counseling program was delivered by a trained midwifery counselor (SH.E.B, MSc in Midwifery) who had completed a 10‐session specialized training course on the GES model and psychosexual counseling. The counselor was supervised throughout the study by a board‐certified psychiatrist (M.H., Associate Professor), who reviewed weekly session summaries and provided guidance on complex cases. In terms of platform usage, each participant exchanged an average of 10–15 messages per week (including group discussion contributions and private chat communications), totaling approximately 50–70 messages over the 6‐week program. These engagement metrics were monitored systematically to ensure consistent participation and adherence. The platform was chosen due to its widespread daily use among Iranian women, its private and encrypted messaging features, and its zero‐cost infrastructure, which together maximized accessibility and cultural acceptability while minimizing stigma and logistical burden.

Educational content included video clips, images, and voice messages, validated by ten faculty members. Each session began with a brainstorming question about participants' lived sexual experiences. Responses were categorized, followed by group consultation and private counseling when needed. Delivery was both synchronous and asynchronous. Weekly assignments (see Table 1) focused on identifying barriers to sexual health (e.g., fears, shame, negative attitudes), enhancing intimacy, and setting flexible sexual goals. Participants shared daily notes and received written feedback from a trained researcher. Women were encouraged to share information with their partners and practice together. The intervention was supervised by an expert psychiatrist. All feedback indicated high engagement and enthusiasm.

## 8 Data Analysis

Data were analyzed using SPSS version 20. Normality was confirmed by the Kolmogorov–Smirnov test (*p *> 0.05). Groups were compared using Chi‐square (or Fisher's exact) for categorical variables and independent t‐tests for continuous variables. Changes across three time points were evaluated using repeated‐measures ANOVA (rANOVA) with group (intervention vs. control) as the between‐subjects factor and time as the within‐subjects factor. Levene's test confirmed homogeneity of variances; sphericity was assessed via Mauchly's test, with Greenhouse Geisser correction applied when violated (Tables 5 and 6). All tests were two‐sided with α = 0.05. Analyses were conducted by a statistician blinded to group allocation. All analyses were prespecified in the registered protocol (IRCT20210926052593N1); no exploratory or subgroup analyses were performed. The study followed CONSORT 2010 [34], Tabachnick and Fidell [35], SAMPL, and the Assel et al. [36] reporting guidelines.

## 9 Privacy and Confidentiality Safeguards

Given the highly sensitive nature of the sexual health topics discussed via WhatsApp, several privacy and confidentiality measures were implemented. First, participants were encouraged to use pseudonyms (nicknames) in the WhatsApp groups; additionally, the research team provided 20 dedicated SIM cards to participants who were unwilling to reveal their personal phone numbers, allowing full anonymity. Second, the research counselor (group administrator) moderated all group discussions, regularly reminded participants not to share identifying information, and had the authority to remove any inappropriate content. Third, participants signed a confidentiality agreement as part of the informed consent process that explicitly prohibited screenshots, forwarding, or sharing of any group content outside the study. The counselor reinforced this rule at the start of each session. Fourth, WhatsApp messages were used solely for counseling purposes and were not retained after the study; all group chats were permanently deleted by the administrator upon study completion. Fifth, all research data (questionnaires) were collected in person at the clinics, not via WhatsApp, and were stored anonymously as coded data with no group identifiers. These measures were approved by the Research Ethics Committee of Urmia University of Medical Sciences (approval No. IR.UMSU.REC.1400.247).

## 10 Results

Of the 168 infertile women screened, 68 were excluded for not meeting the inclusion criteria: 21 lacked internet access, 13 had low literacy, 18 had secondary infertility, 8 were unwilling to participate, and 8 were taking medication for medical issues (see Figure 1 for the flow chart).

Finally, the remaining 100 women were interviewed by the author to fill out the GHQ‐28, Larson's sexual satisfaction and Newton's stress‐related to infertility questionnaires, using the convenience sampling method. Then, they were randomly allocated to the intervention and control groups (50 participants in the control and 50 in the intervention group).

In the next step, WhatsApp groups were created for each group, and the intervention group was given access to the 6–week continuous GES model sexual consultation program. The control group received only routine self‐care advice.

There was no attrition in this study; all 50 participants in each group completed the intervention and follow‐up assessments. This high retention rate (100%) may be attributed to the provision of free SIM cards, continuous personalized follow‐up, and the culturally sensitive, low‐burden design of the WhatsApp‐based intervention. Measurements were taken at three time points: baseline (T0, before intervention), immediately post‐intervention (T1, 6 weeks after baseline), and 1‐month follow‐up (T2, 10 weeks after baseline). Therefore, 50 women in the intervention and 50 women in the control group were followed up and analyzed in the post‐intervention evaluation, and 1 month later (Figure 1).

To investigate the similarity between the groups in terms of socio‐demographic profile and infertility‐related information of the participants, the Chi‐square, trend Chi‐square test, Fisher's exact test, and the independent t‐test were used. There was no significant difference between the intervention and control groups in the mentioned items (all *p* > 0.05). The mean (SD) duration of infertility was 2.66 (1.30) years in the control group and 2.70 (1.28) years in the intervention group. About 14.0% (7/50) of participants in the intervention and 12.0% (6/50) in the control group were housekeepers, and 34.0% (17/50) of participants in both groups had an academic education. (Table 2).

**Table 2:** Comparison of Socio‐demographic Characteristics and Infertility‐Related Clinical Issues of Participants in Control (*n* = 50) and Intervention (*n* = 50) Groups.

Variables	Subcategory	Control *N* (%)	Intervention *N* (%)	Statistic	*p*‐value
Level of education (woman)	Middle school	7/50 (14.0%)	9/50 (18.0%)	X^2^ = 0.017, df = 3	0.80
	High school	9/50 (18.0%)	11/50 (22.0%)		
	Diploma	17/50 (34.0%)	13/50 (26.0%)		
	College	17/50 (34.0%)	17/50 (34.0%)		
Woman's job	Housekeeper	6/50 (12.0%)	7/50 (14.0%)	X^2^ = 0.017, df = 2	0.90
	Self‐employment	33/50 (66.0%)	31/50 (62.0%)		
	Governmental job	11/50 (22.0%)	12/50 (24.0%)		
Husband's education	Elementary	2/50 (4.0%)	2/50 (4.0%)	Fisher's exact test	0.49
	Guidance	9/50 (18.0%)	10/50 (20.0%)		
	High school	9/50 (18.0%)	10/50 (20.0%)		
	Diploma	14/50 (28.0%)	12/50 (24.0%)		
	College	16/50 (32.0%)	14/50 (28.0%)		
Husband's job	Unemployed	2/50 (4.0%)	0/50 (0.0%)	X^2^ = 2.86, df = 4	0.58
	Manual worker	14/50 (28.0%)	12/50 (24.0%)		
	Employee	14/50 (28.0%)	15/50 (30.0%)		
	Shopkeeper	11/50 (22.0%)	15/50 (30.0%)		
	Other	9/50 (18.0%)	8/50 (16.0%)		
Sufficient family income	Enough	20/50 (40.0%)	18/50 (36.0%)	X^2^ = 0.029, df = 2	0.85
	Insufficient	9/50 (18.0%)	11/50 (22.0%)		
	Relatively enough	21/50 (42.0%)	21/50 (42.0%)		
Cause of infertility	Ovarian	28/50 (56.0%)	27/50 (54.0%)	Fisher's exact test	0.98
	Fallopian tube	8/50 (16.0%)	9/50 (18.0%)		
	Endometriosis	5/50 (10.0%)	5/50 (10.0%)		
	Uterine disorders	3/50 (6.0%)	2/50 (4.0%)		
	Thyroid disorders	4/50 (8.0%)	5/50 (10.0%)		
	Other	2/50 (4.0%)	2/50 (4.0%)		

Quantitative variables M (SD)		Control M (SD)	Intervention M (SD)	t (df = 98)	*p*‐value
	Age (years)	28.64 (5.20)	29.20 (5.20)	0.56	0.57
	Frequency of IVF	0.58 (0.85)	0.52 (0.76)	0.36	0.71
	Duration of infertility (years)	2.66 (1.30)	2.70 (1.28)	0.15	0.87

As shown in Tables 3 and 4, the intervention group demonstrated substantial improvements in both primary outcomes immediately after the program and at the 1‐month follow‐up, whereas the control group showed minimal fluctuation over time. These descriptive trends were confirmed by repeated‐measures ANOVA (Tables 5 and 6). The time × group interaction was significant for all outcomes (*p* < 0.001), confirming that the pattern of change over the three time points differed significantly between the two groups. The main effect of time was also significant (*p* < 0.001), indicating an overall change across the study period, while the main effect of group was significant for all outcomes (*p* < 0.001). The effect sizes for the group main effect were large for both primary outcomes (partial η^2^ = 0.52 for total sexual satisfaction and 0.82 for total infertility‐related stress). In essence, the GES intervention led to a marked reduction in infertility‐related stress across all subscales (social, sexual, communication, need for parenthood, and rejection of life without children) and a marked increase in all dimensions of sexual satisfaction (desire, attitude, quality of life, and adjustment), with these improvements largely sustained at the 1‐month follow‐up.

**Table 3:** Descriptive statistics (Mean ± SD) for sexual satisfaction and its subscales at baseline, post‐intervention, and one‐month follow‐up.

Variable	Group	Baseline mean ± SD	Post‐intervention mean ± SD	Follow‐up mean ± SD
Total sexual satisfaction	Intervention	54.94 ± 12.48	96.86 ± 19.48	85.28 ± 17.22
	Control	56.66 ± 15.21	57.55 ± 13.71	57.38 ± 13.98
Sexual desire	Intervention	10.94 ± 2.67	19.28 ± 4.30	17.30 ± 3.90
	Control	11.30 ± 3.45	11.32 ± 3.17	11.01 ± 3.06
Sexual attitude	Intervention	13.34 ± 3.28	23.04 ± 4.74	20.38 ± 4.44
	Control	13.72 ± 4.01	14.12 ± 3.68	14.11 ± 3.87
Sexual quality of life	Intervention	17.46 ± 4.33	31.12 ± 6.43	26.98 ± 5.34
	Control	18.08 ± 4.72	18.66 ± 4.52	18.42 ± 4.67
Sexual adjustment	Intervention	13.20 ± 3.72	23.42 ± 4.71	20.62 ± 4.37
	Control	13.56 ± 4.30	13.78 ± 3.71	13.86 ± 3.51

**Table 4:** Descriptive statistics (Mean ± SD) for infertility‐related stress and its subscales at baseline, post‐intervention, and one‐month follow‐up.

Variable	Group	Baseline mean ± SD	Post‐intervention mean ± SD	Follow‐up mean ± SD
Total infertility‐related stress	Intervention	204.62 ± 16.12	95.72 ± 7.82	154.52 ± 31.85
	Control	205.68 ± 16.18	201.24 ± 18.04	201.91 ± 18.19
Social concerns	Intervention	44.80 ± 4.44	22.76 ± 3.65	33.06 ± 7.11
	Control	45.01 ± 4.63	43.80 ± 4.11	43.94 ± 3.94
Sexual concerns	Intervention	35.70 ± 4.42	17.08 ± 4.42	24.34 ± 7.09
	Control	35.91 ± 4.62	35.38 ± 4.51	35.46 ± 4.63
Communication concerns	Intervention	44.36 ± 4.25	19.74 ± 2.53	35.11 ± 8.65
	Control	44.31 ± 4.21	43.56 ± 4.51	43.68 ± 4.29
Need for parenthood	Intervention	44.66 ± 4.53	19.11 ± 2.58	34.56 ± 7.98
	Control	44.75 ± 4.75	44.18 ± 4.73	44.44 ± 4.56
Rejection of life without children	Intervention	35.51 ± 4.45	17.04 ± 1.94	27.46 ± 6.45
	Control	35.21 ± 4.16	34.32 ± 4.03	34.38 ± 4.16

**Table 5:** Repeated‐measures ANOVA results for sexual satisfaction and its dimensions at three time points: baseline (T0), immediately post‐intervention (T1, 6 weeks), and one‐month follow‐up (T2, 10 weeks) in the intervention and control groups.

Variables	Effect	Source	SS	DF*	MS	F	*p*‐value	Eta
Desire to have sex	Between	Group	1610.1	1	1610.1	98.16	*p* < 0.001	0.50
	Within	Time	932.5	2	466.3	47.07	*p* < 0.001	0.32
		Group × Time	969.4	2	484.7	48.94	*p* < 0.001	0.33
Sexual attitude	Between	Group	1830.3	1	1830.3	84.00	*p* < 0.001	0.46
	Within	Time	1368.7	2	684.4	50.55	*p* < 0.001	0.34
		Group × Time	1148.5	2	574.2	42.42	*p* < 0.001	0.30
Quality of sex life	Between	Group	3468.0	1	3468.0	98.81	*p* < 0.001	0.50
	Within	Time	2659.8	1.88	1418.3	63.89	*p* < 0.001	0.40
		Group × Time	2254.7	1.88	1202.3	54.16	*p* < 0.001	0.36
Sexual adjustment	Between	Group	2144.0	1	2144.0	105.9	*p* < 0.001	0.52
	Within	Time	1466.6	1.85	793.3	44.94	*p* < 0.001	0.34
		Group × Time	1324.9	1.85	716.7	44.60	*p* < 0.001	0.31
Total sexual satisfaction	Between	Group	35381	1	35381	107.9	*p* < 0.001	0.52
	Within	Time	24764	1.88	13202	62.60	*p* < 0.001	0.39
		Group × Time	22138	1.88	11802	55.96	*p* < 0.001	0.36

*Note:* Mauchly's test was used to test sphericity; when violated, the Greenhouse–Geisser correction was applied (as indicated by degrees of freedom < 2 for the within‐subjects effects). In Table 5, SST denotes the Total Sum of Squares.

Abbreviations: DF, degrees of freedom; Eta, Partial Eta Squared; MS, Mean Square; SS, Sum of Squares.

**Table 6:** Repeated‐measures ANOVA results for infertility‐related stress and its dimensions at three time points: baseline (T0), immediately post‐intervention (T1, 6 weeks), and one‐month follow‐up (T2, 10 weeks) in the intervention and control groups.

Variables	Effect	Source	SST	DF*	MS	F	*p*‐value	Eta
Social concerns	Between	Group	8597.4	1	8597.4	365.1	*p* < 0.001	0.79
	Within	Time	6774.7	2	3387.2	149.9	*p* < 0.001	0.61
		Group × Time	5429.9	2	2714.9	120.2	*p* < 0.001	0.55
Sexual concerns	Between	Group	7311.2	1	7311.2	218.9	*p* < 0.001	0.69
	Within	Time	4662.1	2	2331.1	108.5	*p* < 0.001	0.53
		Group × Time	4153.4	2	2076.7	96.67	*p* < 0.001	0.50
Communication concerns	Between	Group	8748.0	1	8748.0	357.8	*p* < 0.001	0.78
	Within	Time	8205.1	1.59	5159.1	152.6	*p* < 0.001	0.61
		Group × Time	7277.2	1.59	4575.7	135.4	*p* < 0.001	0.58
Need for parenthood	Between	Group	10372	1	10372	410.4	*p* < 0.001	0.81
	Within	Time	8793.1	1.78	4929.9	165.5	*p* < 0.001	0.63
		Group × Time	7795.8	1.78	4370.7	146.8	*p* < 0.001	0.60
Rejection of Life without children	Between	Group	8070.4	1	8070.4	179.4	*p* < 0.001	0.65
	Within	Time	1830.5	2	915.2	75.95	*p* < 0.001	0.44
		Group × Time	1788.8	2	894.4	74.22	*p* < 0.001	0.43
Total infertility‐related stress	Between	Group	214775	1	214775	452.3	*p* < 0.001	0.82
	Within	Time	137719	1.76	78075	216.5	*p* < 0.001	0.69
		Group × Time	121167	1.76	68692	190.5	*p* < 0.001	0.66

*Note:* Mauchly's test was used to test sphericity; when violated, the Greenhouse–Geisser correction was applied (as indicated by degrees of freedom < 2 for the within‐subjects effects).

Abbreviations: DF, degrees of freedom; Eta, Partial Eta Squared; MS, Mean Square; SST, Total Sum of Squares.

The effect sizes for the group main effect were large for both primary outcomes (partial η^2^ = 0.52 for total sexual satisfaction and 0.82 for total infertility‐related stress), indicating clinically meaningful improvements according to Cohen's benchmarks (η^2^ ≥ 0.14 for large effects, 0.06 for medium, and 0.01 for small) [37]. Of note, these values represent the group main effect (i.e., the overall difference between the intervention and control groups averaged across all three time points), not the interaction effect (time × group), which was smaller (as shown in the interaction rows of Tables 5 and 6).

Beyond statistical significance, the clinical meaningfulness of the findings deserves emphasis. At post‐intervention, the intervention group showed a mean increase of 41.92 points in total sexual satisfaction from baseline, representing a substantial improvement in a key aspect of quality of life. Although a slight reduction was observed at the 1‐month follow‐up (mean difference from baseline: 30.34 points), scores remained markedly higher than baseline, indicating that the intervention's benefits were largely sustained. Similarly, infertility‐related stress decreased by a mean of 108.90 points immediately post‐intervention, with a 50.10‐point reduction still maintained at follow‐up. These changes, combined with the large effect sizes (partial η^2^ = 0.52 for sexual satisfaction and 0.82 for infertility stress), suggest that the GES program produced improvements that are not only statistically significant but also clinically relevant for this population.

To further assess the precision of the intervention effects, we calculated 95% confidence intervals (CIs) for the key between‐group differences at the primary endpoint (post‐intervention). For total sexual satisfaction, the mean difference between the intervention and control groups was 39.31 (95% CI: 32.14 to 46.48). For total infertility‐related stress, the mean difference was −105.52 (95% CI: −114.38 to −96.66). These narrow confidence intervals indicate that the observed improvements are not only statistically significant but also precisely estimated.

## 11 Discussion

In this randomized controlled trial, we evaluated a WhatsApp‐based sexual counseling program grounded in the Good Enough Sex (GES) model for its effects on infertility‐related stress and sexual satisfaction among infertile Iranian women. The findings demonstrate that the intervention significantly reduced infertility stress and improved sexual satisfaction across all measured dimensions, with large effect sizes for the main effect of group (η^2^ = 0.52 for total sexual satisfaction; η^2^ = 0.82 for total infertility‐related stress). These results support the integration of digitally delivered, theoretically informed psychosexual counseling into routine infertility care, particularly in cultural contexts where face‐to‐face discussion of sexual issues remains highly taboo.

A note of caution is warranted regarding the exceptionally large effect sizes observed in this study (partial η^2^ = 0.52–0.82 for group main effects). Although these values indicate that the intervention produced substantial improvements in both primary outcomes, several factors may have contributed to their magnitude. First, the baseline levels of sexual satisfaction and infertility stress showed considerable room for improvement, creating a potential “floor/ceiling” effect that amplifies observed effect sizes. Second, the high adherence (100% retention, > 90% assignment completion) and the novelty of the intervention in this culturally conservative context where women had never previously had a safe channel to discuss sexual concerns may have produced unusually large effects. Third, the use of partial η^2^ in a repeated‐measures design can yield larger values compared to other effect size metrics, particularly when within‐subject correlations are high. Fourth, the post‐intervention assessment (6 weeks) likely captured peak effects, which may attenuate over longer follow‐up periods. It is also important to note that the effect sizes reported in Tables 5 and 6 represent the group main effect (i.e., the overall difference between intervention and control groups averaged across all time points), not the interaction effect (time × group), which was smaller. Therefore, while these findings are promising, they should be interpreted as preliminary evidence specific to this highly selected sample, and replication in larger, more diverse populations with longer follow‐up is needed before definitive conclusions can be drawn.

Both intervention and control groups were homogeneous regarding socio‐demographic characteristics (age, employment status, education, economic status, and other factors detailed in Table 2). This homogeneity is methodologically important because these variables are well‐established determinants of infertility‐related distress and sexual satisfaction [38, 39, 40, 41, 42]. The absence of baseline differences strengthens the internal validity of our findings and reduces the risk that observed post intervention differences were artifacts of group composition.

Our findings align with and extend previous research on the GES model. Firoozi et al. (2021) demonstrated that face‐to‐face GES counseling improved sexual attitudes and satisfaction among infertile Iranian women [43]. While their work established the model's efficacy in a clinical setting, our study advances this evidence by demonstrating that the same theoretical framework can be effectively delivered via WhatsApp a platform that offers asynchronous, private, and destigmatizing access. In a culture where discussing sexual concerns with a provider in person is often met with shame or fear of moral judgment [11, 14], WhatsApp removes the spatial‐temporal and emotional barriers to help‐seeking. Women who would never utter a word about sexual problems in a clinic waiting room found it possible to type their concerns into a WhatsApp group often using pseudonyms or private messages. This cultural adaptation is not merely a logistical convenience; it is a fundamental mechanism that enabled engagement in the first place.

Similarly, Jamalifar et al. (2022) applied the GES model to women with multiple sclerosis, reporting enhanced intimacy and sexual satisfaction [44]. Our findings reinforce the model's cross‐population efficacy while adding a crucial dimension: infertility‐specific stress reduction. Previous GES studies focused primarily on sexual outcomes; our study is among the first to demonstrate that the model simultaneously alleviates the psychosocial burden of infertility (social concerns, sexual concerns, communication concerns, need for parenthood, and concerns about life without children). This dual benefit is particularly relevant for infertile women, for whom sexual dissatisfaction and general infertility distress are intertwined and mutually reinforcing [45].

Unlike prior digital interventions that required users to access standalone websites or unfamiliar applications [46, 47], our intervention leveraged WhatsApp a platform already deeply embedded in the daily lives of Iranian women. This integration reduced logistical friction and enhanced adherence. More importantly, WhatsApp provided a “psychological safety net” [48]. In a society where sexuality is a stigmatized subject rarely discussed even between spouses [15, 49], the anonymous, text‐based, asynchronous nature of WhatsApp allowed participants to express biological needs and sexual concerns without experiencing direct shame; articulate fears, beliefs, disgust, and negative attitudes that had previously remained unspoken; receive structured psychoeducation without the fear of being judged by a provider face‐to‐face.

Consistent with other studies [48, 50], our findings confirm that digital platforms can destigmatize sexual health conversations by offering anonymity, confidentiality, and user‐controlled pacing. However, our study goes beyond these general observations by embedding platform advantages within an evidence‐based theoretical framework (GES). The combination of WhatsApp's cultural acceptability and the GES model's structured cognitive‐behavioral principles created a uniquely effective intervention for a taboo‐burdened population.

Yazdani et al. (2018) demonstrated the utility of smartphone‐based social networks in improving sexual self‐concept, but their intervention lacked a structured theoretical framework [7]. In contrast, our GES‐based WhatsApp intervention provided participants with a guided, theory‐driven pathway addressing cognitive distortions about sex, unrealistic expectations, and performance pressure. This theoretical grounding likely explains why our intervention achieved significant improvements in both sexual satisfaction and stress reduction, whereas generic social network interventions often report mixed or domain‐specific effects.

A Dutch study successfully implemented an internet‐based sex education program incorporating mindfulness and sensate focus techniques [46]. However, their intervention required participants to actively visit a dedicated website a step that introduces logistical barriers. Our WhatsApp‐based delivery eliminated this barrier by meeting participants in a space they already inhabit daily. This seamless integration is particularly valuable for infertile women, who often face time constraints due to repeated clinic visits, medical procedures, and the emotional labor of managing fertility treatments.

Hucker et al. (2021) demonstrated the efficacy of online mindfulness‐based CBT in improving intimacy among women with sexual disorders [51]. While their approach is therapeutic, ours is psychoeducational and dyadic. For infertile women where relationship dynamics and sexual health education are often neglected guided psychoeducation with partner engagement may serve as a less stigmatizing, more scalable alternative to formal therapy. Our findings suggest that cognitive‐behavioral change can be effectively facilitated through educational empowerment and dyadic skill‐building, without requiring intensive individual therapy.

Van Lankveld's (2016) systematic review established that internet‐based interventions can produce both immediate and sustained improvements in sexual functioning [46]. Our study advances this evidence in three key ways [1]: by targeting infertile women a population with unique psychosexual challenges distinct from general sexual dysfunction [2]; by demonstrating that a theoretically grounded model (GES) can be effectively delivered via a familiar messaging platform; and [3] by showing concurrent improvements in both sexual satisfaction and infertility‐specific stress, addressing the multifaceted nature of sexual health challenges in this population.

Similarly, recent work on online mindfulness programs for endometriosis patients (Hansen et al., 2023) has shown benefits for chronic pelvic pain [52], but our study addresses a distinct population with different etiological and psychological drivers. Infertility‐related sexual distress is driven not by physical pain but by performance pressure, medically timed intercourse, and the social stigma of childlessness all of which the GES model directly targets [53, 54, 55].

In Iranian society, a woman's identity is deeply intertwined with childbearing; infertility is thus experienced not merely as a medical condition but as a fundamental threat to womanhood, often accompanied by feelings of inadequacy, loss of femininity, and identity crisis [16, 17]. Simultaneously, sexuality remains a deeply taboo subject in Iranian culture rarely discussed even between spouses [14, 15]. This dual burden infertility as a threat to feminine identity and sexuality as a forbidden topic creates a “silent suffering” that leaves women without the language or safe space to address their sexual concerns. The WhatsApp‐based GES intervention, by providing an anonymous and private platform, directly addressed this socio‐cultural barrier, offering women a culturally sensitive channel to reclaim their sexual well‐being without violating norms of modesty, while challenging the internalized stigma that equates infertility with personal inadequacy [16]. This cultural framing may partly explain the high engagement and large effect sizes observed in this study.

### 11.1 Strengths

This study has several key strengths. First, it is the first RCT to deliver GES‐based psychosexual counseling via WhatsApp to infertile women in a Middle Eastern context, specifically designed to overcome cultural taboos around face‐to‐face sexual health discussions. Second, WhatsApp‐based counseling offered greater accessibility and economy, allowing participants to contact the counselor anytime, anywhere, while reducing travel costs and time burdens. Third, the platform's anonymity and asynchronicity reduced shame and stigma; many women who had never spoken about sexual issues with anyone including their spouses actively participated. Fourth, the two groups were well matched on all socio‐demographic variables at baseline, minimizing selection bias. Finally, large effect sizes for the main effect of group (η^2^ = 0.52 for sexual satisfaction; η^2^ = 0.82 for infertility stress) indicate clinically meaningful improvements.

### 11.2 Limitations

Several limitations should be considered when interpreting the findings, although they do not invalidate the main conclusions. First, the use of convenience sampling and self‐selection bias cannot be ruled out, as volunteers may have been more motivated or open to discussing sexual concerns. Second, while the anonymous nature of WhatsApp likely reduced social desirability bias, self‐reported measures remain inherently susceptible to over‐ or under‐reporting. Third, the 1‐month follow‐up period precluded assessment of longer‐term sustainability, although the sustained effects observed at 1 month are encouraging. Fourth, the GES model is dyadic in nature, yet we included only women; future research should involve male partners to capture couple‐level dynamics. Fifth, smartphone and internet access were prerequisites for participation, potentially excluding women with lower socioeconomic status or limited digital literacy. Finally, due to the behavioral nature of the intervention, neither participants nor the counselor could be blinded to group assignment, which may introduce performance bias. Nonetheless, the blinded outcome assessment and data analysis minimized detection and analysis biases, and the overall consistency of findings across multiple outcomes strengthens confidence in the results.

## 12 Conclusions

In this trial, WhatsApp‐based GES counseling reduced infertility‐related stress and improved sexual satisfaction among Iranian infertile women, with large effect sizes. Recruitment from two major referral centers in northwestern Iran supports the potential relevance of these findings to the study region, but it does not establish broad regional representativeness. Given the convenience sampling, relatively small sample, restrictive eligibility criteria, and short follow‐up, these findings should be interpreted as promising preliminary evidence. Larger, multicenter trials with more diverse populations and longer follow‐up are needed before routine policy implementation.

## Author Contributions

**Emdadi‐Babanazr Shahla:** investigation, software, writing – original draft. **Moghaddam‐Tabrizi Fatemeh:** conceptualization, investigation, writing – original draft, methodology, validation, visualization, writing – review and editing, supervision. **Haghighi Mahmonir:** conceptualization, methodology, validation, visualization. **Gharaaghaji Asl Rasool:** data curation, software.

## Ethics Statement

This study obtained approval from the Research Ethics Committee of Urmia University of Medical Sciences, Iran (approval No. IR.UMSU.REC.1400.247). All the participants signed written informed consent for their participation in the study. All procedures were performed in accordance with relevant guidelines.

## Conflicts of Interest

The authors declare no conflicts of interest.

## Data Access and Responsibility Statement

All authors have read and approved the final version of the manuscript. Dr. Fatemeh Moghaddam‐Tabrizi (corresponding author) had full access to all of the data in this study and takes complete responsibility for the integrity of the data and the accuracy of the data analysis.

## Transparency Statement

The manuscript guarantor, Dr. Fatemeh Moghaddam‐Tabrizi, affirms that this manuscript is an honest, accurate, and transparent account of the study being reported; that no important aspects of the study have been omitted; and that any discrepancies from the study as planned (and registered with IRCT20210926052593N1) have been explained.

## Data Availability

The data that support the findings of this study are available on request from the corresponding author. The data are not publicly available due to privacy or ethical restrictions.
